# Performance of BIOCREDIT Pf/Pv lactate dehydrogenase-based malaria rapid diagnostic test among pregnant women with suspected malaria infection in Bahir Dar City Administration, northwest Ethiopia

**DOI:** 10.1371/journal.pone.0322362

**Published:** 2025-05-07

**Authors:** Banchamlak Tegegne, Endalkachew Nibret, Abaineh Munshea, Mekonnen Teferi, Mulat Yimer, Getaneh Alemu, Delenasaw Yewhalaw, Dylan R. Pillai

**Affiliations:** 1 Biology Department, Science College, Bahir Dar University, Bahir Dar, Ethiopia; 2 Amhara Public Health Institute, Bahir Dar, Ethiopia; 3 Armauer Hansen Research Institute, Addis Ababa, Ethiopia; 4 Department of Medical Laboratory Science, College of Medicine and Health Sciences, Bahir Dar University, Bahir Dar, Ethiopia; 5 School of Medical Laboratory Sciences, Faculty of Health Sciences, Jimma University, Jimma, Ethiopia; 6 Tropical and Infectious Diseases Research Center, Institute of Health, Jimma University, Jimma, Ethiopia; 7 Pathology and Laboratory Medicine, University of Calgary Cumming School of Medicine, Calgary, Alberta, Canada; Kintampo Health Research Centre, GHANA

## Abstract

**Background:**

Malaria during pregnancy is a common public health problem in sub-Saharan Africa. It poses a double burden as it affects the health of mothers, their fetuses and neonates. Moreover, due to malaria parasites sequestration in the placenta, microscopy might miss infections. Hence, rapid diagnostic tests (RDTs) are good alternatives for diagnosing malaria in pregnancy even at health facilities or periphery or lower-level healthcare facilities (e.g., health posts). However, performance of RDTs should be closely monitored as their sensitivity and specificity are affected by many factors.

**Methods:**

A health facility-based cross-sectional study was conducted among 302 pregnant women with suspected malaria infection to evaluate the performance of the newly introduced BIOCREDIT Pf/Pv plasmodial lactate dehydrogenase (pLDH) RDT. Venous blood samples were collected from all eligible pregnant women and tested for *Plasmodium* infection using BIOCREDIT Pf/Pv and *CareStart*™ Pf/Pv RDTs, microscopy and polymerase chain reaction (PCR) following standard protocols. The performance of BIOCREDIT Pf/Pv was evaluated using the following parameters: sensitivity, specificity, positive and negative predictive values and kappa-value. These parameters were calculated using online SISA software.

**Results:**

Of the 302 pregnant women with complete data, 166 (55.0%), 180 (59.6%), 191 (63.2%) and 207 (68.5%) tested positive by CareStart™ Pf/Pv, BIOCREDIT Pf/Pv, microscopy and PCR, respectively. The sensitivity of BIOCREDIT Pf/Pv in detecting *P. falciparum* was 89.4%, 75.4% and 55.6% as compared to CareStart™ Pf/Pv, microscopy and PCR tests, respectively, while the sensitivity for *P. vivax* was 81.8%, 84.3% and 86.7%, respectively. The specificity of BIOCREDIT Pf/Pv was > 90% when compared to all the three diagnostic tests. When considering PCR as a reference test, BIOCREDIT Pf/Pv was more sensitive (55.6%) than CareStart™ Pf/Pv (37.6%) for detecting *P. falciparum* but had similar sensitivity (86.7%) in detecting *P. vivax*.

**Conclusions:**

BIOCREDIT Pf/Pv performed better for the diagnosis of *P. falciparum* infection in pregnant women than the previously in-use CareStart™ Pf/Pv. We recommend using the BIOCREDIT Pf/Pv RDT in Ethiopia for the diagnosis of both species given the high prevalence and widespread nature of the hrp2/3 gene deletion in the country.

## Background

Malaria is a protozoan disease caused by *Plasmodium* species and transmitted mainly via the bite of female *Anopheles* mosquitoes [1]. According to the World Health Organization (WHO), 2023 report, an estimated 263 million cases and 597,000 deaths due to malaria were reported [2]. Despite malaria affecting all population segments, pregnant women and children under-five are the most vulnerable groups. In 2022, 12.7 million pregnant women were exposed to malaria during pregnancy in the WHO African region [3]. Malaria during pregnancy is associated with several adverse maternal, fetal, and neonatal outcomes such as maternal anaemia, miscarriage, preterm delivery and low birth weight [4,5].

Ethiopia is one of the malaria-endemic countries in Africa, and nearly 52% of the population is at risk of malaria infection [6]. *Plasmodium falciparum* is the predominant species accounting for 70% and *P. vivax* accounting for 30% with rare reports on *P. ovale* and *P. malariae* [1]. Previous reports showed that the Amhara Region of Ethiopia accounted for 31% of Ethiopia’s malaria burden, and 80% of its land mass is receptive to malaria transmission [1,6]. From September to December 2024, a malaria outbreak had occurred in 34 districts of the region [unpublished data].

Based on the recent National Malaria Elimination Guideline of Ethiopia, malaria vector control, surveillance and response, advocacy, communication, social mobilization and early diagnosis and treatment are the four pillars in the malaria control program [1]. Hence, of the malaria control methods, accurate laboratory diagnosis and effective treatment is key in reducing morbidity and mortality [1,7]. In addition, according to the WHO recommendation, all suspected cases of malaria should be diagnosed before treatment [8]. Laboratory diagnosis of malaria can be done routinely using microscopy and rapid diagnostic tests (RDTs), and molecular methods as a confirmatory test. Microscopy and RDTs have been widely used in clinical settings for many years. Even if microscopy is the gold standard test, RDTs are recommended in areas where microscopic examination cannot be performed [9,10]. Moreover, RDTs may be a better diagnostic tool for use in pregnant women, as much of *P. falciparum* sequesters in the placenta and therefore may not be detected on a standard smear, producing false-negative results if diagnosis is based on microscopy alone [11].

Although there are several brands of malaria RDTs available in the market, *P. falciparum*-specific histidine–rich protein 2 (PfHRP2) and species-specific lactate dehydrogenase (LDH) based RDTs are the commonly used target antigens for malaria laboratory diagnosis [10]. However, several factors like improper storage and packaging, poor product design, host immune response and parasitic genetic variation could affect the RDTs’ performance. Moreover, current reports from Ethiopia and the Amhara Region in particular showed that there has been a *pfhrp2* deletion that hampered PfHRP2 laboratory diagnosis for *P. falciparum* [12–15]. The WHO also recommends that the diagnostic strategy needs to be changed from RDTs that exclusively detect HRP2 to non-HRP2, when the prevalence of the *pfhrp2* gene deletion is ≥ 5% [16]. Based on the above evidence, the Ethiopian Ministry of Health switched from HRP2-based RDTs to lactate dehydrogenase (pLDH)-based RDTs targeting the plasmodial lactate dehydrogenase (pLDH) specific for *P. falciparum* and *P. vivax* (RapiGEN BIOCREDIT Malaria Ag Pf/Pv pLDH/ pLDH) at the beginning of the year 2024. Therefore, this study aimed to evaluate the diagnostic performance of this alternative RDT among pregnant women with suspected malaria infection in Bahir Dar City Administration, Amhara Region, northwest Ethiopia.

## Materials and methods

### Study area, design and period

A health facility-based cross-sectional study was conducted from September to November 2024 at Addis Alem Hospital and Bahir Dar Health Center in Bahir Dar City Administration, northwest Ethiopia. According to the Central Statistical Agency of Ethiopia, Bahir Dar City Administration had a total population of 313515, of whom 156870 and 156544 were males and females, respectively. Bahir Dar is located at an altitude ranging from 1722 to 2026 meters above sea level. The city is located approximately 578 km northwest of Addis Ababa and malaria transmission is seasonal, with major and minor transmissions occurring from September to December and April to June, respectively [17].

### Sample size determination

Sample size was calculated using the Buderer’s formula for diagnostic test studies [18].

Study participants: n = (Z^2^ SN (1 − SN))/d^2^

Where n = sample size.

z = 95% statistic for level of confidence (Z = 1.96).

SN = Sensitivity = 75% from WHO recommendation of minimum sensitivity for any malaria RDT [19].

d = margin of error tolerated (d = 0.07).

n = ((1.96)^2^ × 0.75(1 − 0.75))/(0.05)^2^ = 288.

After adding 10% (29) to compensate for non-respondents, a total of 317 pregnant women with suspected malaria infection were recruited for the study.

### Sampling technique

The two health institutions (Addis Alem Hospital and Bahir Dar Health Center) were selected purposively for data collection based on the malaria case flow and proximity to Amhara Public Health Institute, where the laboratory tests (RDT, microscopy and polymerase chain reaction (PCR)) were performed. A sample size of 168 and 134 were proportionally allocated to Addis Alem Hospital and Bahir Dar Health Center, respectively based on the case flow. After estimating (from previous year’s data) the total number of suspected pregnant mothers expected to visit the data collection sites, a sampling interval (k) was calculated, and a systematic random sampling technique was used to select study participants. The first participant was selected by lottery method among the first k pregnant women with suspected malaria infection, and then every kth woman was enrolled in the study. Participants were traced at the antenatal care clinic in both health facilities.

All consenting pregnant women (confirmed by human chorionic gonadotropin hormone test or ultrasound) who fulfilled the clinical case definition for malaria [1] and visited the data collection health facilities during the data collection period were included in the study. Critically ill women who were unable to communicate, those who had taken anti-malaria drugs within four weeks prior to data collection, those who had taken antibiotic or any anti-pain drugs within 24 hours prior to data collection were excluded from the study.

### Sample collection and processing

#### Sample collection.

About 1 ml of venous blood sample was collected from the cubital vein of each participant in EDTA tube following the standard venous blood collection procedure. Samples were transported to Amhara Public Health Institute for malaria diagnosis by microscopy, RDTs and PCR.

#### Microscopic examination.

Both thick and thin blood smears were prepared and labeled properly. After air-drying, thin films were fixed with absolute methanol for five seconds while both thin and thick films were stained with freshly prepared 10% Giemsa working solution diluted with buffered water (PH 7.1–7.2) for 10 min, and examined under 100 × objective. Thick smears were examined to detect *Plasmodium* parasites, while thin smears were used to identify species [20].

#### Malaria diagnosis by RDTs.

It is well known that malaria RDTs diagnose malaria antigens from the blood of infected patients. In this study, *CareStart*™ Pf/Pv (HRP2/pLDH) Ag Combo RDT (USA, RMVM-02591, 2025/02/20) specifically targets PfHRP2 expressed by *P. falciparum* and lactate dehydrogenase specific to *P. vivax* in human whole blood and BIOCREDIT Pf/Pv (pLDH/pLDH) RDT (Republic of Korea, H016D022D, 2026/03/27) targeting the plasmodial lactate dehydrogenase (pLDH) specific for *P. falciparum* and *P. vivax* were used. Manufacturers’ instructions and quality control measures were strictly followed. Briefly, for *CareStart*™ Pf/Pv Malaria RDT, five microliters of blood samples were added using a micropipette to the sample well and two drops (60 μl) of buffer solution were added to the buffer well and results were read just after 20 minutes. For BIOCREDIT Pf/Pv (pLDH/pLDH) RDT, five microliters of blood samples were added using micropipette to the sample well and three drops (90 μl) of buffer solution were added to the buffer well and results were read just after 25 minutes. Negative readings at 25 minutes were read again at 35 minutes.

#### DNA extraction and PET-PCR diagnosis.

*Plasmodium* DNA was extracted using the QIAamp® DNA Blood Mini kit (Qiagen, Hilden, Germany), as recommended by the manufacturer. The photo-induced electron transfer PCR (PET-PCR) assay was run using Agilent Technologies strata gene Mx3005p PCR machine following the protocol described elsewhere [21,22]. The following genus and species level primers were used:

Original Genus 18sFor, 5’-GGC CTA ACA TGG CTA TGA CG-3’

Original Genus FAM 18sRev, 5’-AGG CGC ATA GCG CCT GGC TGC CTT CCT TAG ATG TGG TAG CT-3’

*P. falciparum* For, 5’-ACC CCT CGC CTG GTG TTT TT-3’

*P. falciparum* Rev, HEX-5’-AGG CGG ATA CCG CCT GGT CGG GCC CCA AAA ATA GGA A-3’

*P. vivax* For, 5’-GTA GCC TAA GAA GGC CGT GT-3’

*P. vivax* Rev, HEX-5’- AGG CGC ATA GCG CCT GGC CTG GGG GAT GAA TAT CTC TAC AGC ACT GT-3’

**Fig 1:**
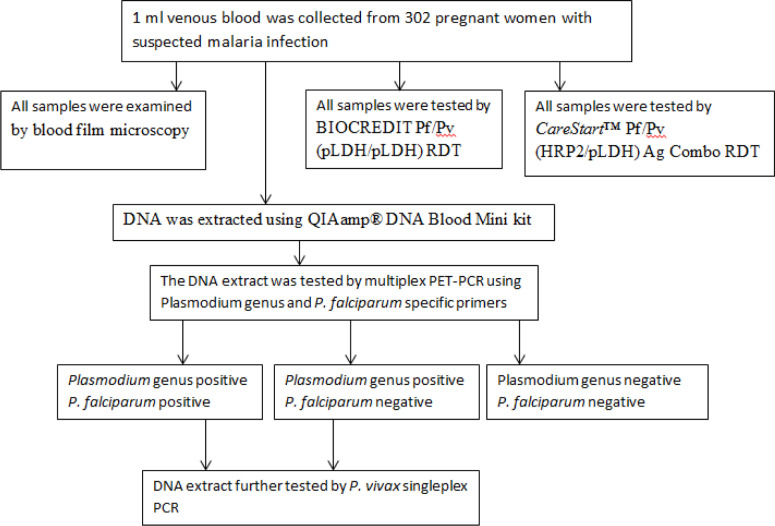
Data collection flow diagram.

Amplification of the *Plasmodium* 18S rRNA gene specific for *Plasmodium* genus, *P*. *falciparum* or *P*. *vivax* was performed in a 20 μl reaction following the protocol explained elsewhere [22]. Samples with a CT value of 40 or below were considered positive [23]. The genus and *P*. *falciparum* multiplex assays were performed for all samples. *P*. *vivax* singleplex assay was performed for genus-positive samples regardless of the result for *P*. *falciparum* (Fig 1).

### Data analysis

Data were entered into Microsoft Excel and sensitivity, specificity, negative predictive value (NPV), positive predictive value (PPV) and kappa coefficient were determined using simple interactive statistical analysis (SISA) online statistical software. Test agreements were declared as no agreement (k ≤ 0), none to slight (k = 0.01–0.2), fair (k = 0.21–0.4), moderate (k = 0.41–0.60), substantial (k = 0.61–0.80) and almost perfect (k = 0.81–1.00) [24].

### Ethical considerations

Ethical approval was obtained from Bahir Dar University, Science College Research Ethical Review Committee (Ref: PRCSVD/814/2023) and permission letter was obtained from Amhara Public Health Institute, Addis Alem Hospital and Bahir Dar Health Center. Written consent was obtained from each pregnant mother who participated in the study. All malaria positive participants were treated with the standard anti-malaria drugs based on the national malaria diagnosis and treatment guideline [1].

### Inclusivity in global research

Additional information regarding the ethical, cultural, and scientific considerations specific to inclusivity in global research is included in the Supporting Information (S1 File).

### Data quality assurance

Data on the hard copy was re-checked after being copied to the excel sheet. For the malaria microscopic examination, slides were read by two trained laboratory technicians independently at the Amhara Public Health Institute. A third WHO-certified laboratory technologist read discordant results between the two technicians. For the malaria RDT diagnosis, standard operating procedures were strictly followed and two laboratory technicians read the results independently. Discordant RDT readings were resolved by discussion. For the PCR assays, we used positive and negative controls in each run. Moreover, the expiry date and storage conditions of reagents and kits were checked before use.

## Results

### Prevalence of *Plasmodium* infection

Of 317 participants initially recruited, samples collected from 302 participants were appropriate for all diagnostic tests and hence included in the data analysis (S2 File). Of 302 samples examined, BIOCREDIT Pf/Pv (pLDH/pLDH) and CareStart™ Pf/Pv (HRP2/pLDH) proved positive in 180 (59.6%) and 166 (55.0%) samples, respectively. Two hundred seven (68.5%) samples tested positive by PET-PCR, whereas 191 (63.2%) samples were positive by microscopy. Results of species-level analysis showed that a higher number of samples tested positive for *P. falciparum* using PET-PCR (117, 38.7%) and the lower using *CareStart*^TM^ Pf/Pv (HRP2/pLDH) (47, 15.6%). Likewise, *P. vivax* was detected in 132 (43.7%) samples with *CareStart*^TM^, followed by PET-PCR (128, 42.4%) and microscopy (122, 40.4%) (Table 1).

**Table1:** Prevalence of *Plasmodium* infection by different diagnostic techniques (N = 302).

*Plasmodium* infection	BIOCREDIT (pLDH/pLDH) RDT N (%)	*CareStart*™ Pf/Pv (HRP2/pLDH) N (%)	Microscopy N (%)	PET-PCR N (%)
*P. falciparum*	67 (22.2)	47 (15.6)	96 (31.8)	117 (38.7)
*P. vivax*	116 (38.4)	132 (43.7)	122 (40.4)	128 (42.4)
Mixed (Pf/Pv)*	3 (1.0)	13 (4.3)	27 (8.9)	38 (12.6)
Total positive	180 (59.6)	166 (55.0)	191 (63.2)	207 (68.5)
Negative	122 (40.4)	136 (45.0)	111 (36.8)	95 (31.5)

*Mixed infections are also included while calculating *P.falciparum* and *P.vivax* prevalence.

**Fig 2:**
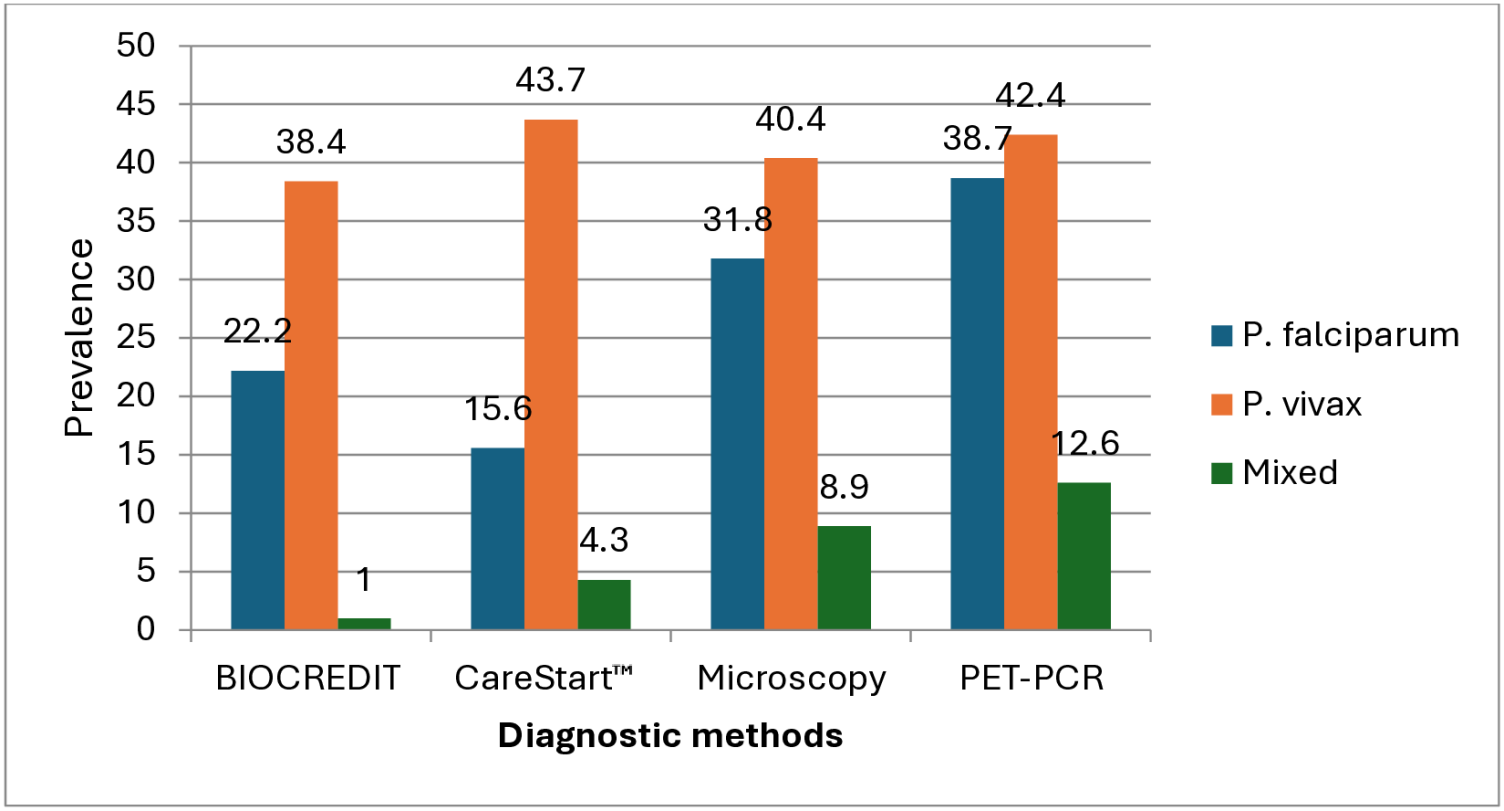
Prevalence of *Plasmodium* species by different diagnostic techniques.

All the four diagnostic tests detected higher number of participants with *P. vivax* mon-infection compared to *P. falcip*arum and mixed infections (Fig 2).

**Fig 3:**
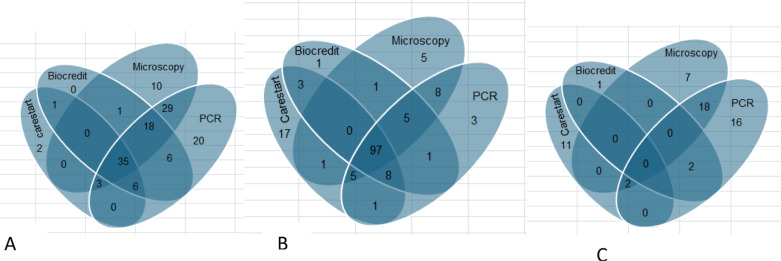
Venn diagram showing detection of *P. falciparum* (A), *P. vivax* (B) and Mixed (C) infections.

A total of 131 (43.4%) and 156 (51.7%) participants were tested positive for *P. falciparum* and *P. vivax* infections, respectively at least by one of the diagnostic tests. Of these, 58 (19.2%) were mixed infections. However, there was variation in the positivity rate among diagnostic tests that only 35 and 97 participants were tested positive for *P. falciparum* and *P. vivax* infections by all diagnostic tests (Fig3).

### Comparison of RDTs with microscopy and PET-PCR

In comparison to PET-PCR and microscopy, BIOCREDIT Pf/Pv RDT misdiagnosed *P. falciparum* in 32 (10.6%) and 54 (17.9%) of the 302 samples, when PET-PCR and microscopy were used as a reference test, respectively. Similarly, BIOCREDIT Pf/Pv RDT misdiagnosed *P. vivax* in 22 (7.3%) and 32 (10.6%) samples, when evaluated against PET-PCR and microscopy reference tests, respectively. When PET-PCR was used as a reference test, *CareStart*^TM^ Pf/Pv (HRP2/pLDH) misdiagnosed *P. falciparum* in 76 (25.2%) and *P. vivax* in 38 (12.6%) samples, respectively (Table 2).

**Table 2:** Comparison of BIOCREDIT Pf/Pv and *CareStart*™ Pf/Pv against microscopy and PET-PCR.

Spp	Results	Biocredit Pf/Pv (pLDH/pLDH)			*CareStart*™ Pf/Pv (HRP2/pLDH)	
CareStart as a reference test N (%)	Microscopy as a reference test N (%)	PCR as a reference test N (%)	Microscopy as a reference test N (%)	PCR as a reference test N (%)
*Plasmodium falciparum*	TP	42 (13.9)	52 (17.2)	65 (21.5)	38 (12.6)	44 (14.6)
	FP	25 (8.3)	15 (5.0)	2 (0.7)	9 (3.0)	3 (1.0)
	TN	230 (76.2)	218 (72.2)	183 (60.6)	197 (65.2)	182 (60.3)
	FN	5 (1.7)	17 (5.6)	52 (17.2)	58 (19.2)	73 (24.2)
*Plasmodium vivax*	TP	108 (35.8)	103 (34.1)	111 (36.8)	103 (34.1)	111 (36.8)
	FP	8 (2.6)	13 (4.3)	5 (1.7)	29 (9.6)	21 (7.0)
	TN	162 (53.6)	167 (55.3)	169 (59.0)	151 (50.0)	153 (50.7)
	FN	24 (7.9)	19 (6.3)	17 (5.6)	19 (6.3)	17 (5.6)

TP = true positive; FP = false positive; TN = true negative; FN = false negative; N = number; % = percentage.

### Performance of BIOCREDIT Pf/Pv and *CareStart*™ Pf/Pv in detecting *Plasmodium* infection

The sensitivity and NPV of BIOCREDIT Pf/Pv (pLDH/pLDH) in detecting *P. falciparum* infection was 75.4% (95%CI: 63.5–85.0) and 92.8% (95%CI: 89.5–95.1), respectively using microscopy as a reference. The sensitivity and NPV were further reduced to 55.6% (95%CI: 46.1–64.7) and 77.9% (95% CI: 74.2–81.2) against a PET-PCR reference. However, BIOCREDIT Pf/Pv (pLDH/pLDH) was more sensitive in detecting *P. falciparum* as compared to *CareStart*™ Pf/Pv (HRP2/pLDH), which had a sensitivity of 39.6% (95%CI: 29.8–50.1) and 37.6% (95%CI: 28.8–47.0) using microscopy and PET-PCR as reference tests, respectively. Hence, BIOCREDIT Pf/Pv was more accurate than *CareStart*™ Pf/Pv (HRP2/pLDH) when evaluated against microscopy (89.4% vs. 77.8%) and PET-PCR (82.1% vs. 74.8%). The sensitivity of BIOCREDIT Pf/Pv and *CareStart*™ Pf/Pv was comparable in the diagnosis of *P. vivax* compared to both microscopy (84.3% vs. 84.4%) and PET-PCR (86.7% vs. 86.7%) reference tests (Table 3).

**Table 3:** Performance of BIOCREDIT Pf/Pv (pLDH/pLDH) and *CareStart*™ Pf/Pv (HRP2/pLDH) in detecting *Plasmodium* infection.

*Plasmodium* infection	Diagnostic tool	Sensitivity (95%CI)	Specificity (95%CI)	PPV (95%CI)	NPV (95%CI)	Accuracy (95%CI)	Kappa value
*Plasmodium falciparum*	Biocredit vs *CareStar*	89.4 (76.9-96.5)	90.2 (85.9-93.6)	62.7 (53.3-71.2)	97.9 (95.3-99.1)	90.1 (86.1-93.2)	0.678
	Biocredit vs microscopy	75.4 (63.5-85.0)	93.6 (89.6-96.4)	77.6 (67.6-85.2)	92.8 (89.5-95.1)	89.4 (85.4-92.6	0.696
	*CareStart*™ vs microscopy	39.6 (29.8-50.1)	95.6 (91.9-98.0)	80.9 (68.0-89.3)	77.3 (74.2-80.0)	77.8 (72.7-82.2)	0.408
	Biocredit vs PCR	55.6 (46.1-64.7)	98.9 (96.2-99.9)	97.0 (89.0-99.2)	77.9 (74.2-81.2)	82.1 (77.3-86.3)	0.591
	*CareStart*™ vs PCR	37.6 (28.8-47.0)	98.4 (95.3-99.7)	93.6 (82.3-97.9)	71.4 (68.4-74.2)	74.8 (69.5-79.6)	0.404
*Plasmodium vivax*	Biocredit vs *CareStar*	81.8 (74.2-88.0)	95.2 (90.9-98.0)	93.1 (87.2-96.4)	87.1 (82.4-90.7)	89.4 (85.4-92.6)	0.782
	Biocredit vs microscopy	84.3 (76.8-90.4)	92.8 (88.0-96.1)	88.8 (82.4-93.1)	89.8 (85.3-93.0)	89.4 (85.4-92.6)	0.778
	*CareStart*™ vs microscopy	84.4 (76.8-90.4)	83.9 (77.7-88.9)	78.0 (71.6-83.3)	88.8 (84.0-92.4)	84.1 (79.5-88.0)	0.674
	Biocredit vs PCR	86.7 (79.6-92.1	97.1 (93.4-99.1)	95.7 (90.3-98.1)	90.9 (86.5-93.9)	92.7 (89.2-95.4)	0.849
	*CareStart*™ vs PCR	86.7 (79.6-92.1)	87.9 (82.1-92.4)	84.1 (77.9-88.8)	90.0 (85.2-93.4)	87.4 (83.1-90.9)	0.743

PPV = positive predictive value; NPV = negative predictive value; PCR = polymerase chain reaction; CI = confidence interval.

## Discussion

To deliver healthcare services at the community level, Ethiopia launched a health extension program in 2004 [25]. One of the program’s main goals has been the prevention and treatment of malaria. As a result, malaria is diagnosed and treated by health extension workers (HEWs) at the lower-level of the healthcare system (e.g., health post) or during house-to-house visits. However, HEWs are not trained to perform malaria microscopy, as there is no laboratory at health posts. Thus, RDTs are used to diagnose malaria. Since both *P. falciparum* and *P. vivax* co-exist in Ethiopia, the country has been using the *CareStart*™ Pf/Pv (HRP2/pLDH), one of the various RDTs currently in use, to diagnose both *P. falciparum* and non-falciparum infections. The diagnosis of *P. falciparum* using *CareStart*™ Pf/Pv (HRP2/pLDH) relies on detecting HRP-2 antigen in the blood. However, recently, reports from several areas of Ethiopia have shown a high prevalence (>5%) of HRP-2/3 gene deletions [12,15,26–29]. In support of this, recent studies in Ethiopia demonstrated that HRP-2-targeting RDTs show low sensitivity [30–32]. Cognizant of this, the Federal Ministry of Health switched from *CareStart*™ Pf/Pv (HRP2/pLDH) to BIOCREDIT Pf/Pv (pLDH/pLDH) at the beginning of 2024.

The prevalence of both *P. falciparum* and *P. vivax* in the present study was high because; (i) data were collected from participants who fulfilled the clinical case definition (i.e., febrile in the last 48 hours and coming from malaria endemic area) [1], (ii) data were collected during the major malaria transmission season (September to December), (iii) there was malaria epidemics in the study area at the time of data collection, and (iv) we used sensitive molecular diagnostic test (PET-PCR), in addition to microscopy and RDTs. In Ethiopia *P. falciparum* is known to account for 70% of malaria infections, while the remaining 30% are due to *P. vivax*, but this figure may vary by season and locality [1]. Hence, the prevalence of *P. vivax* was higher than that of *P. falciparum* in the present study (Table 1). This is supported by recent literature reporting a 7-fold national increase in *P. vivax* malaria cases from 100,000 in 2018–730,000 in 2022, unlike the steady increase in *P. falciparum* cases [33]. Similarly, a higher prevalence of *P. vivax* than *P. falciparum* was reported in a previous study in Ethiopia [34]. The shift in the proportion of the two species is multifactorial, with climate change and relapse might being primary contributors [35]. A recent report suggested the expansion of *P. vivax* populations that could infect both Duffy-positive and Duffy-negative individuals in sub-Saharan Africa, including Ethiopia, contributing to the increasing proportion of *P. vivax* compared to *P. falciparum* [36]. However, to provide a definitive justification, large-scale studies are recommended to assess the role of vectors, human factors, the environment and climate-related drivers contributing to the distribution of each *Plasmodium* species.

The present study revealed that, compared to *CareStart*™, the BIOCREDIT Pf/Pv RDT showed good performance in detecting *P. falciparum*, with only 5 false-negative (FN) results compared to 25 false-positive (FP) results. This was inconsistent with a prior study in Djibouti [37]. The higher FP rate might be due to HRP2/3 gene deletions missed by the *CareStart*™ but detected by the BIOCREDIT RDT [12–15]. The BIOCREDIT RDT was also more sensitive than the previously used *CareStart*™ in detecting *P. falciparum* when evaluated against microscopy (75.4% vs. 39.6%) and PCR (55.6% vs. 37.6%) reference tests, which was in line with a previous study from Burundi [38]. However, BIOCREDIT RDT’s sensitivity (55.6%) against PCR was less than the WHO’s minimal cut-off value (≥75%) [19]. Additionally, it fell short of sensitivity rates observed in other studies, such as 79.9% in Burundi [38], 88.2% in Djibouti [37], 87.8% in Uganda [39], 69% [40], and 89% in Ethiopia [41]. These discrepancies may be due to factors such as parasite densities, RDT manufacturers, storage and transportation conditions, and operator skills [42–45]. Although we did not quantify parasite density in this study, we anticipated that most individuals had minimal parasitemia because the study area was under a malaria elimination program. Hence, 5.6% FN results were reported compared to PCR (Table 2). This might have occurred due to the higher detection limit of malaria RDTs (>100 parasites/µl) compared to that of the reference PCR test (<5 parasites/µl) [46]. The impact of operator skill on RDT performance was demonstrated in Djibouti, where retraining and close supervision increased the sensitivity to identify *P. falciparum* from 69.8% to 88.2% (p < 0.01) [37].

The high specificity (98.9%) and PPV (97.0%) were substantiated by the fact that, when compared to PCR, only two samples had FP results with BIOCREDIT. This was consistent with earlier findings [37,39,47]. When compared to microscopy, the FP was higher (5.0%), which could be because of the limited sensitivity of microscopy itself (5–20 parasites/µl compared to a detection limit of < 5 parasites/µl by PCR) [46,48]. Moreover, *P. falciparum*-infected red blood cells are sequestered in the placenta. So, some *P. falciparum* infections might have been overlooked by microscopy but detected by the BIOCREDIT RDT. It is also crucial to remember that individuals with rheumatoid factor [49] or those with persistent pLDH following treatment [50] could have experienced BIOCREDIT FP results.

When compared to PCR, the BIOCREDIT RDT’s sensitivity (86.7%) and NPV (90.9%) for detecting *P. vivax* were above the minimum sensitivity limit of RDTs [19], but lower than findings of earlier studies [37,47]. The sensitivity was similar to that of the *CareStart*™ (86.7%), implying that either RDT could be recommended for the diagnosis of *P. vivax* infections in Ethiopia. However, the BIOCREDIT RDT is more appropriate as both *P. falciparum* and *P. vivax* are co-endemic in most parts of the country [1].

In the present study, *CareStart*™ (13 samples) detected higher number of mixed infection compared to BIOCREDIT (3 samples). Differences in cross-reactivity, and hence specificity between the two RDTs might bring this variation. For instance, as explained in the manufacturers’ leaflets, *CareStart*™ produces a false positive result for patients with acute schistosomiasis. Schistosomiasis is highly prevalent in the study area. Biocredit is 100% specific in detecting *P. vivax* than *Carestart* having a specificity of 97.5% (source: manufacturers’ leaflets), possibly contributing for the higher report of P. vivax mono-infection as well as mixed infection by *Carestart*. However, it needs further investigation to give definitive justification.

This was the first study to assess the performance of the newly introduced BIOCREDIT Pf/Pv RDT in Ethiopia. We believe the findings of this study could provide valuable information regarding the performance of the BIOCREDIT Pf/Pv RDT in detecting both *P. falciparum* and *P. vivax* in Ethiopia. Nevertheless, the current study had some shortcomings: (i) parasite densities were not quantified, and the impact of parasitemia level on RDT performance was not assessed; (ii) sociodemographic and pregnancy-related data were not collected to evaluate the role of factors on RDT performance; (iii) RDT tests were performed only by the investigators, and the impact of operational skill by the HEWs (who regularly perform RDT tests) was not assessed; and (iv) subclinical (asymptomatic) participants, who usually carry low parasite loads, were not included in the study, so we are unable to recommend the use of BIOCREDIT *Pf*/*Pv* RDT for mass screening.

## Conclusions

The BIOCREDIT Pf/Pv RDT demonstrated superior performance in diagnosing *P. falciparum* compared to the previously in-use *CareStart*™ Pf/Pv (HRP2/pLDH). For diagnosing *P. vivax,* its performance was comparable to that of *CareStart*™. We recommend the use of the BIOCREDIT Pf/Pv RDT for diagnosing *Plasmodium* infections in areas with a high prevalence of *hrp2/3* deletions in Ethiopia. However, comprehensive nationwide study is recommended to evaluate the performance of BIOCREDIT Pf/Pv RDT in various malaria transmission settings.

## Supporting information

S1 File Ethical, cultural, and scientific considerations specific to inclusivity in global research. (DOCX)

S2 File Raw data. (XLSX)

## Abbreviations

CT: Cycle Threshold
FMoH: Federal Ministry of Health
FN: False Negative
FP: False Positive
HEW: Health Extension Workers
HRP-2: Histidine-Rich Protein 2
LDH: Lactate Dehydrogenase
NPV: Negative Predictive Value
PCR: polymerase chain reaction
PET-PCR: Photo-Induced Electron Transfer Polymerase Chain Reaction
pLDH: Plasmodial Lactate Dehydrogenase
PPV: Positive Predictive Value
RDT: Rapid Diagnostic Test
TN: True Negative
TP: True Positive
WHO: World Health Organization.
